# ZoteroBib

**DOI:** 10.5195/jmla.2020.1084

**Published:** 2020-10-01

**Authors:** Tara J. Brigham

**Affiliations:** 1 brigham.tara@mayo.edu, Assistant Professor, Medical Education, and Librarian, Mayo Clinic Libraries, Mayo Clinic, Jacksonville, FL

## Abstract

**ZoteroBib.** Corporation for Digital Scholarship, 8300 Boone Boulevard, Suite 500, Vienna, VA 22182; https://zbib.org/; pricing: free.

## GENERAL DESCRIPTION

ZoteroBib is a free, web-based citation generator from the same team that developed Zotero. ZoteroBib, or Zbib, was released in 2018 and is maintained by the Corporation for Digital Scholarship (also known as “Digital Scholar”). The Corporation for Digital Scholarship is a nonprofit organization founded in 2009 that is dedicated to the development of software and services for researchers and cultural heritage institutions [1]. While it might be easy to initially confuse Zotero and ZoteroBib, the main difference lies in what each tool can do and who uses it.

Zotero is a citation/reference manager and is useful software for almost anyone who is doing serious, long-term research [2]. ZoteroBib is a citation generator for those who create occasional bibliographies or individuals who are interested in teasing out metadata for quickly citing a resource. This review focuses primarily on the features of ZoteroBib, with a brief comparison to the other existing tools.

## FEATURES

### Accessibility

All that is required to use ZoteroBib is a device and an Internet connection. No mobile app is available, but it is optimized for tablet and phone use. This resource is compatible with any browser and does not require users to download software or create an account. The bibliography is stored in the browser's local storage, so these data remain entirely under the user's control [3]. If users are in private or ?incognito mode, their bibliographies will be deleted when they close their browser windows.

### Usability

Using ZoteroBib is easy and straightforward: users insert an identifier—uniform resource location (URL), International Standard Book Number (ISBN), digital object identifier (DOI), PubMed identifier (PMID)—or the title of the citation into the search bar. ZoteroBib will then automatically grab data from “newspaper and magazine articles, library catalogs, journal articles, sites like Amazon and Google Books, and much more” [4]. If the automatic import finds incomplete data or does not find any data for a reference, a manual editor can be used to create the citation.

If the citation is entered manually, ZoteroBib enables the user to choose the type of citation, with typical options such as book or journal article, and allows choices like artwork, podcast, or statute. The entry fields change based on the citation type. After the citation is created, it is added to the “Bibliography” section below the search bar.

Each citation can be manually edited in the bibliography, and the citation style can be altered at any time. ZoteroBib supports more than 9,700 citation style languages, including widely used styles from the American Psychological Association (APA) and Modern Language Association (MLA). In addition, ZoteroBib has styles commonly used in medicine like American Medical Association (AMA), Vancouver, National Library of Medicine (NLM), and those used for specific medical journals like the *New England Journal of Medicine* and *Nature*. Because ZoteroBib is connected with Zotero, all of the styles are kept up to date with current style guide recommendations and publisher requirements. For example, when APA released the eighth edition of their style guide, Zotero updated from APA 7 to APA 8, and the style format was updated in ZoteroBib.

Once the user is satisfied with the bibliography, the options for copying or exporting the bibliography are:

- Copy to Clipboard (copy citations to a local computer's clipboard)
- Download rich text format (RTF) (download option for all word processors)
- Copy hypertext markup language (HTML)
- Download RIS
- Download BibTex
- Save to Zotero

Another helpful option that ZoteroBib offers is the ability to link to the current collection of citations that users have gathered. The “Link to this version” option generates an URL that can be used to retrieve that version of a bibliography at a later date. The “Link to this version” URL is useful if the bibliography is loaded onto another computer or shared. The shared bibliography is generated as a read-only version but can be loaded into the editor if changes need to be made.

ZoteroBib has an FAQ web page that contains information separated into three areas: General, Usage, and Troubleshooting [4]. If specific help or errors are located, these can be reported to Issue/Ask for Assistance in the Zotero Forums [5]. Requesting support from Zotero (@zotero) on Twitter also seems to work for any problems with using ZoteroBib.

## BRIEF COMPARISONS

One of the most noticeable differences between ZoteroBib and other citation generators is that ZoteroBib presents a “clean” interface without advertisements. Most citation generators are littered with bothersome ads, which are distracting for anyone trying to use these websites. Some of the other citation generator tools might also require that users create accounts to access premium features, such as additional citation styles or export features. This can be cumbersome for those who just want a quick, but reliable way to get an accurate reference.

Another advantage ZoteroBib has over other citation generators is it can generate bibliographies in more than 9,700 different styles [3]. This feature is especially useful for those in the health sciences. While most of the free citation generators have the APA citation style available, they may lack the ability to generate citations in AMA, Vancouver, NLM, or other popular medical citation styles.

In this reviewer's opinion, the main disadvantage to ZoteroBib is when a citation does not have an identifier or pulls the wrong information from a URL or a DOI, the data have to be entered manually; however, as mentioned above, ZoteroBib will guide the user in entering the necessary information, based on the type of reference. Another drawback is that ZoteroBib does not allow the user to change the order of the citations in the bibliography if the style dictates that the references are numbered. Although the user can delete and correct the order of citations in the numbered bibliography, it is unfortunate that amending the order of the numbered references is not an option.

Finally, although perhaps not as necessary for health sciences librarians, ZoteroBib does not appear to provide as many alerts to users to be cautious about untrustworthy references. Websites like myBib and EasyBib both encourage users to consider the credibility of a citation and remind users to look for certain information to be included (e.g., author, date of creation for a website entry) [6, 7].

Table 1 provides an overview of the various features of different citation generators.

**Table 1:** Overview of features of different citation generators

	Pulls metadata for each citation with only entering one identifier (e.g., DOI, ISBN)	No ads on website/Does not require purchasing/ creating an account for more functionality	Ease of use/No coding or programming experience needed	No download required	Exports in multiple formats†	Generates bibliographies in multiple citation styles‡	Generates easily copied citations in multiple citation styles†	Generates in-text citations in multiple citation styles†	Helps check credibility of citation§
ZoteroBib	Y	Y	Y	Y	Y	Y	Y	Y	N
AMA Citation Generator (Mick Schroeder)	Y	Y	Y	Y	N	N	N	N	N
Auratikum	Y	N	Y	Y	UTA	UTA	UTA	UTA	UTA
BibGuru	Y	Y	Y	Y	Y	N	N	N	N
Bibliograph	Y	Y	N	Y	Y	UTA	UTA	UTA	UTA
Bibliography.com	Y	Y	Y	Y	N	N	N	N	N
BibMe*	Y	N	Y	Y	N	N	N	N	Y
Citace PRO	Y	N	Y	Y	N	UTA	UTA	UTA	UTA
CitationGenerator	N	Y	Y	Y	N	N	N	N	N
Citation Machine*	Y	N	Y	Y	N	N	N	N	Y
Citationsy	Y	N	Y	Y	UTA	UTA	UTA	UTA	UTA
Cite4Me	N	N	Y	Y	UTA	UTA	UTA	UTA	UTA
Cite This For Me*	Y	N	Y	Y	N	Y	Y	Y	N
Citefast	Y	N	Y	Y	N	N	N	N	Y
Crossref	Y	Y	Y	Y	Y	N	N	N	N
DOI Citation Formatter	Y	Y	Y	Y	N	N	Y	N	N
EasyBib*	Y	N	Y	Y	N	N	N	N	Y
Formatically	N	Y	Y	Y	N	N	N	N	N
Google Scholar	Y	Y	Y	Y	Y	N	Y	N	N
Harvard Generator	N	N	Y	Y	N	N	N	N	N
KnightCite v3.1	N	Y	Y	Y	N	N	N	N	Y
MyBib	Y	N	Y	Y	UTA	Y	Y	Y	Y
ReFindit	Y	Y	Y	Y	N	N	Y	N	N
ResearchoMatic	N	N	N	Y	UTA	UTA	UTA	UTA	UTA
Sciwheel (formerly, F1000Workspace)	Y	N	UTA	Y	UTA	UTA	UTA	UTA	UTA
Scribbr	Y	N	Y	Y	N	N	N	N	N

Y=Yes; N=No; UTA = Unable to assess.

* Chegg owns the following websites or web citation tools, and they all function essentially the same way: BibMe, Citation Machine, Cite This For Me, and EasyBib.

† Multiple export formats=more than just exporting to MS Word or GoogleDoc.

‡ Multiple citation styles=more than just American Psychological Association (APA), Modern Language Association (MLA), Chicago Manual of Style/Turabian, and Harvard styles.

§ The website/tool either had to provide feedback on credibility of citation information or remind the user of what to look for in a trustworthy resource.

## PRACTICAL USAGE

ZoteroBib can be used in the daily life of a health sciences librarian to:

- show undergraduate students how to easily create a bibliography if they do not need to build an ongoing database of citations
- generate and export a list of detailed citations from grey literature sources that do not offer an easy export option of their metadata for a systematic review
- assist nursing students in how to properly cite their references in APA format
- help a frugal researcher with exporting all of their citations using PubMed Central identifiers (PMCIDs), but without any hidden or unreadable code that could interfere with the particular formatting needed in a grant application
- generate and export citations of US legal documents or law information for a psychiatrist's manuscript

## SUMMARY

ZoteroBib is a free and easy-to-use tool for working with references. While it is not designed to be used for managing references for long-term research projects, it can help everyone from undergraduates to senior researchers quickly generate citations and bibliographies. It is a handy resource for anyone working in health sciences libraries.
